# A New IL-6-Inducing Mechanism in Cancer with New Therapeutic Possibilities

**DOI:** 10.3390/cancers16213588

**Published:** 2024-10-24

**Authors:** Leif Håkansson, Pontus Dunér, Erik Broströmer, Bengt Gustavsson, Yvonne Wettergren, Bijar Ghafouri, Annika Håkansson, Birgitta Clinchy

**Affiliations:** 1 Division of Clinical Tumorimmunology, Department of Oncology, University Hospital of Linkoping, 581 85 Linkoping, Sweden; 2 Therim Diagnostica AB, 236 37 Höllviken, Sweden; 3 Department of Clinical Sciences Malmö, Lund University, 205 02 Malmö, Sweden; 4 Ström & Gulliksson AB, P.O. Box 793, 220 07 Lund, Sweden; 5 Department of Surgery, Institute of Clinical Sciences, Sahlgrenska Academy, University of Gothenburg, 405 30 Gothenburg, Sweden; 6 Department of Surgery, Region Västra Götaland, Sahlgrenska University Hospital, 413 45 Gothenburg, Sweden; 7 Pain and Rehabilitation Center, Department of Health, Medicine and Caring Sciences, Linköping University, 581 85 Linköping, Sweden; bijar.ghafouri@liu.se; 8 Department of Oncology, Uppsala University Hospital, 751 85 Uppsala, Sweden; 9 Department of Clinical and Experimental Medicine, Division of Clinical Immunology, Linköping University Hospital, 581 85 Linköping, Sweden

**Keywords:** interleukin-6, cancer, PBMCs, immunoregulation, albumin neo-structure, IL-6-inducing factor, autoantibodies, immune complexes, prognosis

## Abstract

**Background**: Interleukin-6 is dysregulated in multiple pathological conditions, e.g., cancer and inflammatory diseases. **Aim**: To investigate new mechanisms for the regulation of pathological IL-6 production. **Methods**: PBMCs (peripheral blood mononuclear cells) stimulated by cancer serum factors or specific peptides produce interleukin-6 (IL-6). Immunoregulatory albumin neo-structures and peptides were identified with 2D gel electrophoresis and MALDI-TOF-MS (matrix-assisted laser desorption/ionization time-of-flight mass spectrometry) analyses. Il-6 and albumin neo-structures were determined by ELISA (enzyme-linked immunosorbent assay). **Results**: Conformational changes in normal serum albumin by proteolytic degradation generates an IL-6-inducing neo-structure, IL-6-inducing factor (IL-6IF). This neo-structure is immunogenic which results in the production of autoantibodies. IL-6 production induced by IL-6IF and cancer patient sera is inhibited by specific antibodies. The serum concentration of IL-6IF is significantly higher in advanced cancer stages, and its presence is significantly correlated with the survival of the patients. **Conclusions**: A new mechanism for the induction IL-6 synthesis is presented. Based on this mechanism, the pathological IL-6 production related to enhanced proteolytic activity can be diagnosed and selectively inhibited by specific antibodies. Such antibodies were identified and purified. Thus, the neo-structure, inducing pathological IL-6 production, associated with a reduced survival of cancer patients, can be selectively removed by the therapeutic administration of antibodies leaving the function of IL-6 needed for the normal activity of the immune system intact.

## Simple Summary

Cytokines are substances important in the regulation of the immune system. The cytokine interleukin-6 (IL-6) is important for the normal function of the immune system and also plays a pathogenic role in multiple diseases such as chronic inflammation, autoimmunity cancer, sepsis, and cardiovascular disease. A new immunoregulatory mechanism is described. Proteolytic degradation of serum albumin generates immunoregulatory neo-structures, one of which has the capacity to induce IL-6, Il-6-inducing factor, IL-6IF. The serum concentration of this factor is significantly increased in advanced stages of colon cancer and is associated with cancer-specific survival of the patients. IL-6IF is immunogenic, resulting in the production of autoantibodies with the capacity to inhibit IL-6IF-induced IL-6 synthesis. The specificity of these autoantibodies, using the single B-cell technique, enables the production of human monoclonal antibodies, which therapeutically can be used to specifically inhibit pathological IL-6 production, leaving IL-6 for the normal function of the immune system intact.

## 1. Introduction

Interleukin-6 (IL-6) is a pleiotropic cytokine of major importance for human health and well-being. Due to its signal transduction mechanisms, it has the capacity to modulate the activity of numerous types of cells in various organs [1,2]. It is thereby involved in the pathogenesis of a large number of different conditions such as chronic inflammatory and autoimmune diseases, cancer, severe infections, cardiovascular disease, the metabolic syndrome, type 2 diabetes, and neurodegenerative diseases. These conditions are usually characterized by the overproduction of IL-6 with an enhanced serum concentration.

Different types of cells have the capacity to produce IL-6. Inducing mechanisms, post-translational regulation, and the “The Local Initiation Model” were recently reviewed [3]. In addition to cytokines and prostaglandins [3], the cross-linking of Fc receptors [4] plays a regulatory role.

IL-6 plays a fundamental role in the cytokine network and is pivotal for initiating a normal immune response. However, it is also a central player in immune system dysregulation, in over-activation, as seen in inflammatory diseases and cytokine storm, or in immunosuppression as in chronic inflammation and cancer [5,6]. In addition, it has the capacity to promote tumor progression by stimulating angiogenesis, metastasis, and proliferation of tumor cells [7]. Furthermore, it acts as an autocrine growth factor in some malignancies, e.g., myeloma and renal cell carcinoma. The binding of IL-6 to its receptor results in the activation of the signal transducer and activator of transcription 3 (STAT3) [8]. The activation of STAT3 is of central importance in many resistance mechanisms [9,10] involving chemotherapy, immunotherapy, and radiotherapy [11]. In particular, the blockade of IL-6 is suggested to overcome resistance to check-point inhibitors [12,13].

A meta-analysis of the IL-6 serum concentration and prognosis in more than 11,000 patients with 23 different types of cancer in 100 studies concluded that the IL-6 serum concentration correlates with the prognosis in later stages, independent of cancer type [14]. In this analysis, the results on colorectal cancer were, however, considered to be inconclusive. In a systematic review on gastrointestinal cancer, a high serum concentration of IL-6 was found to correlate with a poor prognosis in gastric, bile duct, and pancreatic cancers, but not in colorectal cancer [15]. However, in the meta-analysis by Xu et al., it was concluded that a high serum concentration of IL-6 was associated with poor prognosis in colorectal cancer [16].

Tissue expression of IL-6 in colorectal cancer correlated with lymph node metastasis, venous invasion, and an advanced stage and was a poor prognosis predictor [17,18]. Similarly, tumor expression levels of IL-6 and IL-6 receptor (IL-6R) were prognostic factors for over-all survival and metastasis-free survival in soft-tissue sarcoma patients [19]. Tumor expression of IL-6 was also found to be a marker of poor prognosis in cervical cancer [20,21]. However, tumor-infiltrating interleukin-6-positive immune cells at the invasive front were associated with significantly longer survival in early-stage colorectal cancer patients [22].

PBMCs co-cultured with ovarian cancer cells from advanced patients were found to produce significantly more IL-6 compared to PBMCs co-cultured with early-stage cancer cells [23]. IL-6 synthesis by PBMCs from patients with hepatocellular carcinoma [24] and breast cancer [25] was found to correlate with prognosis. To further increase the diagnostic efficacy by analyzing the production of IL-6 in PBMC cultures, a very low concentration of Lipopolysaccharide (LPS) was added to cell cultures from colorectal cancer patients. Indeed, patients with lymph node metastases and normal IL-6 production had a good prognosis, whereas patients with such metastases and an enhanced production of IL-6 were found to have progressive disease [26]. Similar results using potentiation by LPS were also reported for pancreatic cancer [27] and head and neck squamous cell carcinoma [28]. The dysregulation of the immune system was further explored by analyzing the signaling response to IL-6 [29] or IFN-γ [30] by PBMCs from breast cancer patients. The IL-6 results in both these studies correlated with the prognosis.

Several signal transduction pathways have been described for IL-6, such as classic signaling where IL-6 binds directly to its receptor, IL-6R, and then to gp130; cluster signaling where the IL-6-IL6R complex on one cell binds to gp130 on another cell; and trans-signaling where a complex of IL-6 and soluble IL-6R binds to gp130 [31].

Gp130 is widely expressed on various cells, the function of which can then be modulated. Interestingly, soluble IL-6R and gp130 in the blood can function as a buffer for IL-6 serving as a protection against overstimulation [2]. Classic signaling induces anti-inflammatory and reparative activities, whereas trans-signaling is proinflammatory. The latter mechanism is involved in several inflammatory conditions, and its blockade by sgp130fc (Olamkicept) has a therapeutic activity [2].

Thus, IL-6 plays a fundamental role not only for the normal function of the immune system, but also for its dysregulation. These diverse functions complicate the possibility to use IL-6 as a therapeutic target in conditions where disease control at least partly depends on the normal function of the immune system [32]. In rheumatoid arthritis and Castelman’s disease, the inhibition of IL-6 significantly contributes to disease control. Cytokine storm related to CAR T-cell treatment in blood malignancies is efficiently inhibited by IL-6 blockade [33]. In cancer, IL-6 inhibitors have been described to counteract various pro-tumorigenic activities, but clinical cancer control in humans has not been reported. Siltuximab, a monoclonal antibody binding to and inhibiting the function of IL-6, as a single drug, did not achieve cancer control as shown in multiple studies on various solid tumors [34,35,36]. The lack of effect in these cancers might be due to the fact that in some of these patients, the immune system is still of importance for disease control. In addition, the blockade of IL-6 carries the risk of infectious diseases as observed during Siltuximab treatment of multiple myeloma [37,38,39].

Although IL-6-mediated trans-signaling can be selectively inhibited by sgp130fc, the uncontrolled overproduction of IL-6 in pathological conditions is still not fully understood [1]. It would thus be important to find initiators of IL-6 production specific for pathological conditions leaving the normal regulation of IL-6 intact.

In the search for new and so far unexplored immunosuppressor mechanisms in cancer, it was found that albumin neo-structures generated by proteolytic fragmentation had immunoregulatory activities such as the stimulation of IL-6 synthesis. This mechanism is further described in the present paper.

## 2. Material and Methods

### 2.1. Preparation of Peripheral Blood Mononuclear Cells

Venous blood was drawn in glass vacuum tubes with acid dextrose citrate solution A as anti-coagulant (Vacutainer, Becton Dickinson, Franklin Lakes, NJ, USA). PBMCs were isolated by Ficoll-Paque Plus (GE Healthcare Bio-Sciences AB, Uppsala, Sweden) density gradient centrifugation after removal of erythrocytes by sedimentation in 2% dextran T500 solution (Amersham Pharmacia Biotech AB, Uppsala, Sweden) in 0.9% NaCI. PBMCs were suspended in RPMI/2% human serum albumin (HSA), and the cell concentration was adjusted to 5 × 10^5^ lymphocytes/mL.

### 2.2. Preparation of Serum Samples

Sera (collected in Vacutainer, Becton Dickinson, Franklin Lakes, NJ, USA) were heat-inactivated for 30 min at 56 °C and frozen at −70 °C. After thawing, sera were diluted in RPMI1640 to a concentration of 20% and used in co-culture experiments for cytokine induction with control PBMCs. A sample of 100 μL of diluted sera (20%) or ultrafiltered serum fractions was added to microtiter plates together with 100 μL PBMC suspension (5 × 10^5^/^mL^). Cell-free supernatants (SNs) were harvested after over-night incubation and tested for monokine activity by ELISA.

### 2.3. Ultrafiltration

Filtrates from 100,000 mw (molecular weight) filters, retentates or filtrates from 50,000 mw filters, and fractions concentrated on a 3000 mw cut-off filter were used (Amicon Centriplus centrifugal filters, Millipore Co., Burlington, MA, USA). Retentates were reconstituted in RPMI1640 with 200 IU/mL penicillin, 200 μg/mL streptomycin, and 4 mM L-glutamine (Sigma, Saint Louis, MO, USA) to their original volume.

### 2.4. Preparation of Urine Samples

Urine samples from renal cell carcinoma or malignant melanoma patients or healthy individuals were centrifuged for 10 min at 3000× *g* followed by filtration with a 0.45 μm Millex-HV syringe filter (Millipore, Burlington, MA, USA) and filtered at 50 000 mw and concentrated on a 3000 mw filter. Before co-culture with normal PBMCs, the samples were buffer-exchanged to RPMI1640 with 200 IU/mL penicillin, 200 μg/mL streptomycin, and 4 mM L-glutamine (Sigma) by gel filtration over a Sephadex-G25 (PD-10) desalting column (Pharmacia, Uppsala, Sweden).

### 2.5. Albumin Peptides

Synthetic albumin peptides, >95% pure, were prepared by Schafer-N (Copenhagen, Denmark). They were reconstituted in sterile water (Sigma) for use in ELISA or in RPM11640 (GIBCO, Waltham, MA, USA) and sterile-filtered for use in cell cultures.

### 2.6. Generation of Cell Culture Supernatants for Cytokine Determination

A sample of 100 μL of culture medium consisting of RPMI1640 supplemented with 200 IU/mL penicillin, 200 μg/mL streptomycin, 4 mM L-glutamine (Sigma Chemical, Saint Louis, MO, USA), and 20% fresh heat-inactivated autologous serum was added to microtiter plates followed by 100 μL of PBMC suspension (5 × 10^4^ lymphocytes) in RPMI/2%HSA. PBMCs from healthy donors were also cultured in synthetic AIM V Medium (Thermo Fisher, Waltham, MA, USA). In some experiments, lipopolysaccharide (LPS, Sigma Chemical Co., Saint Louis, MO, USA) was added at a concentration of 0.05 ng/mL. SNs were harvested after 24 h, and residual cells were removed by centrifugation (refrigerated Beckman, Brea, CA, USA) at 2600× *g* for 5 min. SNs were frozen and stored at −70 °C until cytokine concentrations were measured by ELISA.

### 2.7. Cyto-/Monokine ELISA

Cytokines in culture supernatants were assessed by ELISA using the DuoSet ELISA development system for human IL-6 (R&D Systems Europe Ltd., Abingdon, UK) following the manufacturer’s recommended procedures. Samples were analyzed as mean of triplicate wells.

### 2.8. Autoantibody ELISA

The ELISA Maxisorp 96-well plate (Nunc; #442404, ThermoFisher, Stockholm, Sweden) was coated with a solution of 1× phosphate-buffered saline (PBS), pH 7.4, containing 5 μg/mL Peptide-IL-6IF at 100 μL per well. The plate was incubated overnight at 4 °C. The next day, healthy and cancer patient sera/plasmas were defrosted at 37 °C for 30 min, then diluted to 2% in a solution of PBS containing 1% fish gelatin (G7041, Merck-Sigma-Aldrich, Stockholm, Sweden) as a buffering and blocking agent (FG-PBS). The 2% diluted samples were heated at 56 °C for 30 min. The ELISA plate was washed and blocked and 100 μL per well of 2% diluted serum or plasma was added to relevant wells. The plate was incubated for 2 h at room temperature, and the detection antibody, a horseradish peroxidase (HRP)-conjugated sheep-anti-human-IgG antibody (NA933, GE; Merck-Sigma-Aldrich, Stockholm, Sweden) diluted 1/3000 in FG-PBS, was added at 100 μL per well. The plate was incubated for 60 min at room temperature, and TMB developer solution (Sigma; #T4444) was added to the wells (100 μL), and the plate was incubated for 30 min at room temperature in darkness. The development was stopped with 100 μL per well of 1 molar sulfuric acid. Absorbance readings were taken at 450 nm and 570 nm in a FluoStar Omega plate spectrophotometer (BMG LabTech, Orrtenberg, Germany).

### 2.9. Inhibition ELISA

Healthy sera were defrosted at 37 °C for 30 min, then diluted to 2% in a solution of PBS containing 10% Superblock in PBS solution (Thermo Scientific; #37515). The 2% samples were heated at 56 °C for 30 min in a water bath in order to release autoantibodies (anti-modified protein antibodies). Pierce™ Protein G Coated 96-well plates were then coated with 0.2% autoantibody solution in PBS-T and blocked with 10% Superblock in PBS. Diluted (100 μg/mL) probes (biotinylated IL-6IF peptide) ± inhibition with serum factors in 10% Superblock in PBS-0.1% Tween solution were added and incubated at 37 °C during 60 min. Rabbit anti-Streptavidin-Alkaline Phosphatase, AKP (diluted 1:1000) in Superblock 1:10 in PBS-0.1% Tween was added (100 µL/well) and incubated for 60 min at room temperature (RT). PNPP Substrate was dissolved in diethanolamine Substrate Buffer (1×), and 100 µL was added per well. Samples were incubated for 30–90 min at RT. The color development was monitored; the absorbance was measured at 405 nm, and the reference wavelength at 570 nm was used to compensate for plate impurities.

### 2.10. Adsorption of Human Sera with Protein G-Coupled Sepharose

Heat-inactivated human serum was incubated with protein G Sepharose (Merck-Sigma-Aldrich, Stockholm, Sweden. A total of 20% original serum or 20% protein G non-binding serum was added to the cell culture medium.

### 2.11. Sample Preparation for 2D Gel Electrophoresis

Urine samples (100–450 mL) from cancer patients or healthy controls were ultra-centrifuged on Jumbosep centrifugal devices (Pall Life Science, Ann Arbor, MI, USA) using a 30 K membrane insert or, alternatively, with a Proflux M12 system using a 30 K Pellicon 2 mini filter (Millipore, Burlington, MA, USA) followed by concentration on Jumbosep with a 3 K membrane insert. The urine fraction, 3–30 KD, was tested for cytokine-inducing activity as previously described.

### 2.12. PBMC-Adsorbed Urine Fractions from Cancer Patients

Cytokine production by the PBMCs in response to urine fractions from cancer patients was verified.

Approximately 50 × 10^6^ PBMCs were added to 2.7 ultrafiltered (3–30 KD) urine fractions pooled from two patients with renal cell carcinoma. Unabsorbed urine fractions, used as controls, received the equivalent volume of PBS without PBMCs. The urine fractions were incubated for 1½ h at 4 °C. The adsorbed urine fractions were tested for cytokine-inducing activity.

### 2.13. 2D Gel Electrophoresis and Mass Spectrometry

Two-dimensional gel electrophoresis was performed in a horizontal 2D set-up (Multiphore/IPGphore, Pharmacia Biotech, Uppsala, Sweden) as described based on isoelectric focusing (IEF) in the first dimension and molecular mass in the second dimension [40]. Briefly, samples (230 µg, 350 µg, and 600 µg) were applied to IPG gels, pH 4–7, (Amersham Pharmacia Biotech, Uppsala, Sweden) and focused overnight for 48,000 Vh. SDS-PAGE was then carried out with 16% T/1% C polyacrylamide-casted slab gels. Molecular weight standards were included in each run. Separated proteins were detected by Coomassie blue staining or SYPRO ruby staining. The protein patterns in the gels were analyzed as digitized images using a CCD (charged-coupled device) camera (1340 × 1040 pixels) in combination with a computerized imaging 12-bit system, PDQuest Version 6.1.0 (Bio-Rad Laboratories, Solna, Sweden, in the case of fluorescent stained gels using UV scanning illumination mode (Fluor-S Multi-imager, Bio-Rad Laboratories, Solna, Sweden). The amount of protein in a spot was assessed as background-corrected optical density, integrated over all pixels in the spot, and expressed as integrated optical density (IOD). Tryptic digests of excised protein spots were performed using MALDI_TOF MS (Voyager-DE PRO, Applied Biosystems, Foster, CA, USA) as previously described [41].

Electrotransfer and N-terminal sequence analyses: Selected protein spots were electrotransferred to PVDF membranes (Bio-Rad Labboratories, Solna, Sweden) and subjected to N-terminal sequence analysis by Edman degradation in a Procise cLC or a Procise HT sequencer (PE-Applied Biosystems, ThermoFisher Scientific, Stockholm, Sweden) at the Protein Analysis Center, Karolinska Institute, Stockholm, Sweden.

### 2.14. Fragmentation of IgG and Serum Albumin Using Matrix Metalloproteinases (MMPs)

MMP-1, -2, and -13 (R&D Systems) and MMP-3 and -7 (Merck-Sigma-Aldrich, Chemicon, Stockholm, Sweden) were activated according to instructions of the manufacturer and were incubated with 1 mg/mL of either human serum albumin (HSA, Octapharma, Stockholm, Sweden) or pooled human IgG (IvIg, Gammagard, Baxter, Søborg, Denmark) The mixtures were incubated for 5–20 h at 37 °C and buffer-exchanged to RPMI and tested for cytokine inducing activity.

### 2.15. Fragmentation of IgG and Serum Albumin Using Homogenized Tumor Biopsies

Frozen human tumor biopsies from patients with renal cell carcinoma, malignant melanoma, or colon carcinoma were suspended in 3–10 mL cold RPMI/PEST (Penicillin Streptomycin) or PBS/PEST and homogenized using a Mikro-Dismembrator U (B. Braun Biotech International, Melsungen, Germany). The tissue was transferred to a PTFE shaking flask together with 1 mL RPMI/PEST or PBS/PEST and a tungsten carbide grinding ball and homogenized during 15–20 s with a shaking frequency of 1500–2000 RPM. The homogenized tumor tissue with 20 mg/mL HSA in RPMI/PEST was incubated for 18–21.5 h at 37 °C. The supernatants were buffer-exchanged to RPMI and tested for cytokine-inducing activity with normal PBMCs.

### 2.16. Identification of Immune Cell-Binding Albumin Sequences—Artificial Cell Surface Chromatography

PBMCs were prepared from 4 × 450 mL blood and suspended in PBS containing Ca and Mg (GIBCO, ThermoFisher Scientific, Stockholm, Sweden) at a concentration of 10 × 10^6^/mL. EZ Link Sulfo-NHS-biotin (Pierce, ThermoFisher Scientific, Stockholm, Sweden) was added at a final concentration of 0.2 mg/mL, and the mixture was incubated on a shaker at room temperature for 10 min. Biotinylated PBMCs were lysed by adding 1.0 mL ice-cold lysing buffer (50 mM Tris-HCL, pH 7.5, with 0.15 M NaCI, 5 mM MgCI_2_ containing 100 mM Octyl glucoside (Merck-Sigma-Aldrich, Stockholm, Sweden) and 1 mM phenylmethylsulfonyl fluoride (Merck-Sigma-Aldrich, Stockholm, Sweden)) per 2 × 10^7^ pelleted cells with gentle shaking, then incubated for 30 min on ice. Debris was removed by centrifugation.

### 2.17. Preparation of Affinity Column with Biotinylated Cell Surface Proteins from Mononuclear Cells Coupled to Streptavidin Sepharose

Biotinylated cell lysate, corresponding to 36 × 10^7^ mononuclear cells, in lysate buffer was diluted 1/10 in binding buffer (20 mM NaH_2_PO_4_, 0.15 M NaCI, pH 7.5) It was added to a 1 mL Hitrap Streptavidin HP affinity column (Amersham Biosciences, Amersham, UK). The column was carefully washed with PBS and stored in PBS with 0.1% NaN_3_ at 4 °C until use.

### 2.18. Proteolytic Fragmentation of Denatured Human Serum Albumin with Trypsin

Freeze-dried HSA (0.5 mg) was reconstituted in 25 mM NH_4_HCO_3_, pH 8, containing 10 mg sequencing grade modified trypsin (Promega Corporation, Fitchburg, WI, USA) and incubated at 37 °C overnight. Unfragmented albumin and enzyme were removed by ultrafiltration. The filtrate, containing fragmented HSA without enzyme was collected and diluted with PBS with Ca and Mg (GIBCO).

### 2.19. Adsorption of Trypsin-Fragmented dHSA Using an Affinity Column with Biotinylated Cell Surface Proteins (ACS)

Two ml of trypsin-fragmented dHSA in PBS, corresponding to a total of 0.2 mg protein, was passaged over the ACS column. The flow-through was collected in small portions of 0.2 mL. Thirty µL of each sample, including a control sample that had not been adsorbed, was dried in a Speed-Vac centrifuge.

### 2.20. Mass Spectrometry

Dried samples were reconstituted in 10 µL of 0.1% TFA. Zip Tip pipette tips (Saint Louis, MO, USA) containing C_18_ reversed-phase media were used for desalting reconstituted samples. For analysis of samples in the mass range 700–3600 Da, one μL of each Zip Tip-eluted sample was mixed with 1 μL of a saturated solution of α-cyano-4-hydroxycinamic acid (0.02 mg/mL) in 70%acetonitrile/0.3% trifluoro acetic acid (Merck-Sigma-Aldrich, Stockholm, Sweden). For the analysis of samples in the mass range 1500–9000 Da, 1 μL of each Zip Tip-eluted sample was mixed with 1 μL of sinapinic acid (3-methoxy-4-hydroxycinnamic acid) (Merck-Sigma-Aldrich, Stockholm, Sweden). A sample of 1 μL of the mixture was spotted on the MALDI plate and analyzed using MALDI-TOF MS (Voyager-DE PRO, Applied Biosystems, CA, USA). Mass identity search for resulting spectra was performed in the SwissProt or NCBI database using MS-Fit.

### 2.21. Generation of Rabbit Antiserum Specific for Albumin Peptide IL-6IF

Peptide IL-6IF was synthesized and conjugated with keyhole limpet hemocyanin (Schafer-N, Copenhagen, Denmark). Polyclonal antisera were generated by immunizations of rabbits with the conjugate and Freund’s adjuvants (Agrisera AB, Umeå, Sweden). Rabbit antibodies were prepared using affinity chromatography over protein-A Sepharose and columns with bound IL-6IF peptide (Ultralink lodoacetyl gels (Pierce Biotechnology Inc., Rockford, IL, USA). For cell cultures, buffer was exchanged to RPMI and sterile-filtered.

### 2.22. Statistical Analysis

Data were analyzed with the GraphPad Prism v10 or Excel v16.85. Comparisons of the means between different patient groups or different test occasions were performed using an unpaired *t*-test or Mann–Whitney test. Comparisons between multiple groups were analyzed with one-way ANOVA + Dunnett’s multiple comparisons test. Pearson’s Correlation test was used to analyze serum IL-6 concentrations and autoantibodies. Time to progression and survival were analyzed using the Kaplan–Meier method and Logrank test. Standard error of the mean (SEM) or standard deviation (SD) were used. Specific test method is indicated in figure legends.

## 3. Results

### 3.1. Production of IL-6 by PBMCs

In the search for a model to further explore regulation of IL-6 synthesis, PBMCs from cancer patients were found to produce large amounts of IL-6 in short-term cultures with autologous serum in the medium (Table 1). In a first series, patients with radically resected stage III melanoma and untreated patients with metastatic melanoma or renal cell carcinoma were compared to healthy controls, and in a second series, primary colorectal cancer patients were compared with such controls.

The serum concentration of IL-6 in these patients was generally below the detection limit of the ELISA technique used. Thus, it is likely that serum IL-6 levels are not only derived from the tumor but are also produced to a considerable degree by PBMCs. This means that mononuclear blood cells in vitro are somehow induced to produce the cytokine. Furthermore, IL-6 production is not restricted to patients with advanced disease as PBMCs from patients with primary colorectal cancers and radically resected stage III melanomas (MM 0) also showed enhanced production.

### 3.2. Occurrence and Characterization of a Serum Factor Inducing IL-6 Production by PBMCs

The production of IL-6 by PBMCs from cancer patients in vitro means that either the producing cells were triggered in vivo and maintained this status in cultures or that there is a serum factor which stimulates the production of IL-6. Sera from cancer patients with a high IL-6 production in autologous cultures induced IL-6 in cultures with normal PBMCs in five out of five cultures (Table S1, patients 1–5) demonstrating the presence of an IL-6-inducing serum factor. In contrast, sera from patients who did not produce IL-6 in autologous cultures (patient 6–7) did not induce IL-6 in healthy control PBMCs (Table S1).

### 3.3. Characterization of IL-6IF in Serum and Urine by Ultrafiltration

Ultrafiltration of sera revealed that Il-6-inducing activity generally has a molecular weight of less than 50 kD, in some sera of more than 50 kD, but it was not found in the less than 3 kD fraction. These results indicate either that the IL-6-inducing activity can depend on molecules of different sizes or that a low-molecular-weight factor is bound to other serum proteins. If the IL-6IF is a small fragment, it is likely that it is bound to other serum proteins or cells as it would otherwise be excreted in the urine. IL-6-inducing activity was actually found in the 3–50 kD fraction in urine from five out of five patients. Urine from two healthy controls had only low IL-6-inducing activity.

### 3.4. Identification of Interleukin-6-Inducing Activity

**Figure 1:**
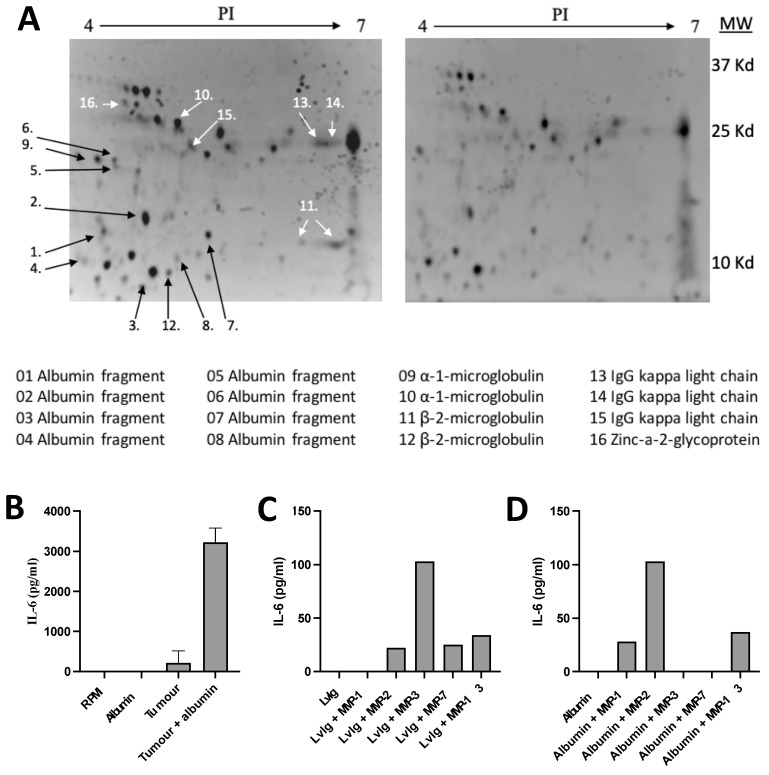
Identification of immune cell-binding protein fragments and IL-6-inducing albumin neo-structures. (**A**) Two-dimensional gel electrophoresis of pooled urine fractions (3–30 kD) from two patients with renal cell carcinoma; unabsorbed urine is shown to the left and the fraction adsorbed by purified normal PBMCs to the right. Adsorbed proteins/fragments are identified by numbers, and their identity is shown at the bottom of the Figure. (**B**) Generation of IL-6-inducing factors by incubation of serum albumin with tumor homogenate. (**C**) Generation of IL-6-inducing factors by incubation of IvIg with activated MMPs, in particular, MMP-2, -3, -7, and -13 released active fragments from IgG. (**D**) Generation of IL-6-inducing factors by incubation of serum albumin with activated MMPs, in particular, MMP-1, -2, and -13 released active fragments from albumin.

The nature of IL-6-inducing factors was further explored by identifying protein fractions in cancer patient urine. Urine fractions with the capacity to produce high amounts of IL-6 were adsorbed by incubation with a surplus of mononuclear blood cells. The IL-6-inducing activity was thereby significantly reduced. Protein fractions in unabsorbed and adsorbed urine were then compared in 2D gel electrophoresis. A reduction in the amount of the proteins after adsorption was recorded visually and confirmed using densitometry (Figure 1A).

Adsorbed proteins were identified using MALDI-TOF MS or amino acid sequencing according to the Edman technique (nine proteins). The identified proteins are shown at the bottom of Figure 1A. The majority of these fractions were found to be fragments of normally occurring proteins, such as serum albumin, immunoglobulins, and microglobulins, indicating that IL-6-inducing substances might be in the group of albumin fragments with a molecular weight below 25 kD or immunoglobulin-related fragments with a molecular weight below 37 kD.

These fragments were identified based on their binding to PBMCs, indicating the occurrence of receptors for these protein fragments on normal PBMCs sensitive to IL-6-inducing factors. As normal albumin does not bind to these cells and induce IL-6, it can be anticipated that the fragmentation of albumin results in conformational changes exposing new structures, neo-structures, with a specific binding to receptors on PBMCs.

### 3.5. Production of IL-6-Inducing Activity by Adding Serum Albumin to Washed Tumor Homogenates or Serum Albumin or IgG to Activated Matrix Metalloproteases (MMPs)

Tumor homogenates were thoroughly washed to make sure that they did not contain any IL-6-inducing activity. Such homogenates or activated MMPs were incubated with serum albumin or IgG in order to study if tumor tissue (Figure 1B) or MMPs (Figure 1C,D) have the capacity to generate active fragments from these proteins.

It was then found that the addition of IgG and albumin markedly increased the production of IL-6-inducing activity. The cytokine-inducing activity was analyzed in cultures with normal PBMCs, and IL-6 production was determined using the ELISA technique. These results indicate that IL-6-inducing fragments of serum albumin or IgG can be produced in the intra-tumoral milieu.

### 3.6. Identification of the IL-6-Inducing Albumin Sequence—IL-6IF

The results described above indicate that albumin fragments have the capacity to induce production of IL-6. Therefore, a more systematic search for immunoregulatory albumin sequences was started. An artificial cell surface (ACS) was prepared by selectively biotinylating cell surface structures of PBMCs. The cells were dissolved, and the biotinylated proteins were bound to streptavidin columns. A mixture of peptides obtained after the trypsination of albumin was adsorbed on such ACS columns. The binding peptides were then identified by using the MALDI TOF MS technique. One of the identified peptides, a 24 mere close to the C-terminal of albumin, peptide IL-6IF, was found to induce the production of IL-6 when added to PBMCs from healthy individuals in short-term cultures.

### 3.7. Programming of PBMCs In Vivo

Increasing amounts of albumin were added to PBMC cultures, and supernatants were collected after 24 h. The amount of albumin neo-structure was determined using ELISA where plates were coated with rabbit antibodies directed to the IL-6IF structure. Interestingly, more of these albumin neo-structures were generated in PBMC cultures from advanced cancer patients compared to healthy controls (Figure S1). This indicates that there is an enhanced amount of the IL-6-inducing neo-structure present in cancer patient sera and that such neo-structures are produced selectively in PBMC cultures from cancer patients, probably due to a unique set-up of proteases.

### 3.8. Immunogenicity of Albumin Neo-Structures—Autoantibodies (Anti-Modified Protein Antibodies) Against the Neo-Structure IL-6IF

**Figure 2:**
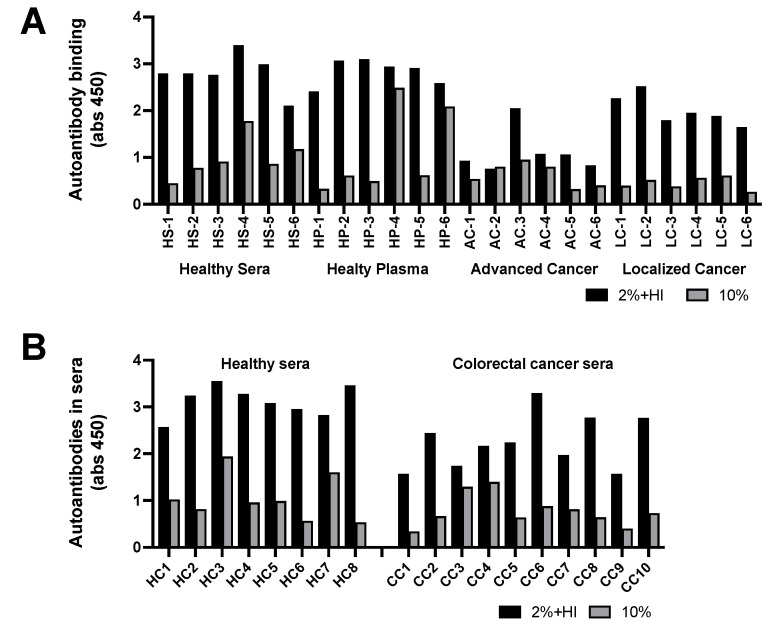
Occurrence of autoantibodies directed against IL-6IF. (**A**) Occurrence of autoantibodies directed against the IL-6IF structure in sera or plasma from healthy controls and advanced or localized cancer patients. Serum and plasma from 6 controls were compared, and the antibody titers were found to be very similar. The antibody titers in sera from one group with advanced, metastatic cancer and one with localized head and neck cancer are shown. Grey bars, samples diluted to 10% and black bars, samples diluted to 2% and heat-inactivated for 30 min at 56 °C. Sera from healthy controls (HS 1–6), plasma from healthy controls (HP 1–6), sera from advanced cancer (AC 1–6), and sera from localized cancer (LC 1–6). (**B**) Occurrence of autoantibodies against the IL-6IF structure in sera from a group of colon cancer patients compared to healthy controls. Sera from healthy control samples (HC 1–8) and sera from preoperative colon cancer samples (CC 1–10).

Immunoregulation by albumin neo-structures is further complicated as some of these neo-structures including IL-6IF are immunogenic, resulting in the production of autoantibodies (Figure 2). The presence of both the antigen, that is IL-6IF, and the autoantibodies (anti-modified protein antibodies) in vivo results in the development of immune complexes. Consequently, the albumin neo-structures and autoantibodies exist in immune complexes and as free antigens and antibodies depending on their ratio. In order to enable proper determination and clinical evaluation, the constituents of these complexes have to be further characterized.

Using the ELISA technique, autoantibodies in sera were bound to plates coated with the IL-6IF peptide followed by the determination of antibodies using an anti-IgG antibody.

The analyses of two types of samples are shown in Figure 2A,B. Serum or plasma was diluted to 10% (grey bars) or diluted to 2% and heat-inactivated at 56 °C for 30 min (black bars). It was found that standard heat inactivation of plasma or sera resulted in the release of a large number of free antibodies, presumably due to the aggregation of albumin neo-structures, which block antibody binding sites, resulting in a release of autoantibodies. Interestingly, the antibody titer against IL-6IF in sera diluted to 2% (five times more) and heat-inactivated contained significantly more free antibodies than sera just diluted to 10% in both the healthy controls and in patients with localized cancer (*p* < 0.0001) but not in patients with advanced cancer, indicating the presence of IL-6IF in a high concentration in advanced disease. This is also demonstrated by the fact that the serum titer of such autoantibodies after heat inactivation was significantly lower in advanced cancer (*p* < 0.0001) or localized cancer (*p* < 0.0002) compared to healthy controls, indicating an increased elimination of immune complexes due to a high production of the antigen. An additional cohort of samples from patients with colon cancer is shown in Figure 2B. The serum titer of IL-6IF autoantibodies after heat inactivation was significantly lower in colon cancer compared to healthy controls (*p* < 0.0016). The heat inactivation released significantly more autoantibodies in both the healthy control and in cancer serum (*p* < 0.0001).

### 3.9. Effect of Antibodies on IL-6 Synthesis Induced by the IL-6IF Peptide or Serum Factors

**Figure 3:**
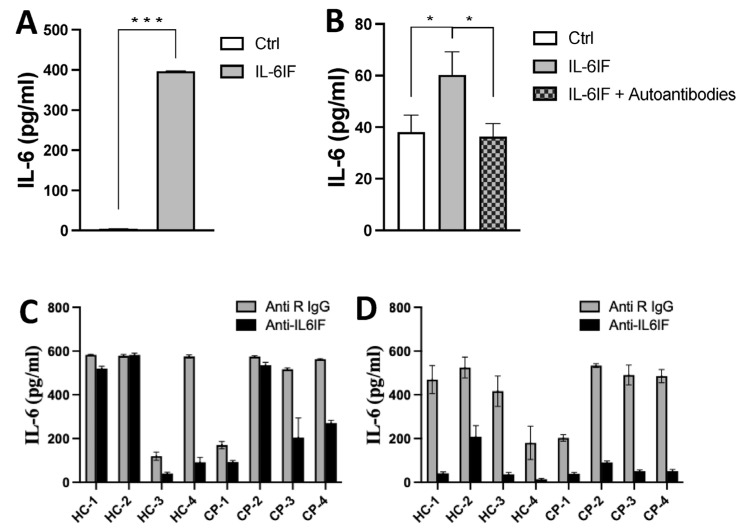
IL-6 synthesis induced by IL-6IF or serum factors and its inhibition by autoantibodies or rabbit antibodies directed against this factor. (**A**) Production of IL-6 in short-term cultures of PBMCs from healthy individuals after addition of the IL-6IF peptide (albumin sequence 512–536). The concentration of IL-6 in culture supernatants after 24 h was measured using the ELISA technique. Bars represents mean ± SD, *** *p* < 0.001, *t*-test. (**B**) Inhibition of the IL-6-inducing activity of peptide IL-6IF by affinity-purified autoantibodies in PBMCs cultured with synthetic medium without serum. Bars represents mean ± SD, * *p* < 0.05, *t*-test. (**C**) IL-6 production by control PMBCs in the presence of healthy or cancer sera diluted to 10% with addition of LPS, 0.05 ng/mL. Rabbit IgG or anti-IL-6IF antibodies at a concentration of 20 µg/mL were added to half of the cultures (C). Sera from healthy control samples (HC) 1–4 and cancer patient samples (CP) 1–4. (**D**) Same experimental set-up as (**C**) but after depletion of sera by protein G beads, the effect of the anti-IL-6IF antibody is enhanced.

The IL-6IF peptide has the capacity to induce IL-6 synthesis by PBMCs (Figure 3A). In another experiment, in order to avoid any influence by serum factors, the cultures were set up with a synthetic medium (Figure 3B). The IL-6 synthesis was efficiently inhibited by incubation of the inducing medium with affinity-purified autoantibodies directed against the IL-6IF structure bound to protein G beads (Figure 3B). This result strongly supports the regulatory effect of autoantibodies on IL-6 synthesis in vivo.

The impact of IL-6IF structures in sera on IL-6 synthesis was further explored in PBMC cultures where cells from healthy donors were exposed to sera from cancer patients or healthy donors. Rabbit antibodies directed against the IL-6IF were added to the cultures in order to inhibit the IL-6IF structure in the sera (Figure 3C,D).

These antibodies had no effect on IL-6 synthesis in cultures with sera from two controls and one cancer patient (Figure 3C). IgG and immune complexes can have a regulatory effect on IL-6 production by PBMCs [4]. Therefore, IgG and CIC were, in another set of cultures, removed from the sera by adsorption using protein -G Dynabeads. This procedure significantly enhanced the inhibitory effect of specific rabbit antibodies blocking the IL-6IF structure (Figure 3D) in all cultures indicating that cross-linking of the Fc receptor (FcR) or cross-linking of the FcR and the cellular receptor of IL-6IF might play an important role in the regulation of IL-6 synthesis. As the antibodies in this experiment significantly reduced IL-6 production, the IL-6IF structure is the main inducing factor and LPS, at this concentration, plays a minor role for IL-6 synthesis.

### 3.10. Tumor Stage Influences IL-6 Production by PBMCs from Colorectal Cancer Patients

**Figure 4:**
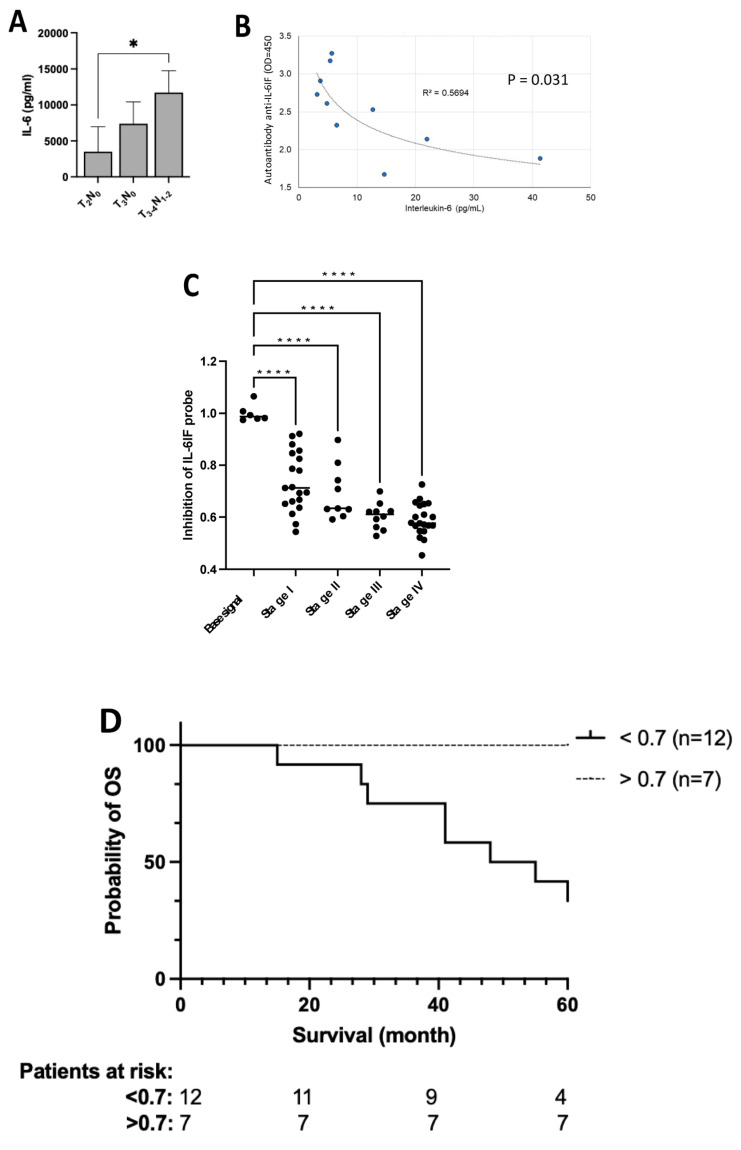
IL-6 correlation to cancer stage and serum concentration of autoantibodies and association of serum concentration of IL-6IF and cancer stage and cancer-specific survival. (**A**). Impact of tumor burden on IL-6 production by PBMCs from colorectal cancer patients. Even patients with localized disease have significant production of IL-6. Bars represents groups (mean ± SD), * *p* < 0.05 (Mann–Whitney). (**B**) Correlation between the serum concentration of autoantibodies directed against IL-6IF structures and the serum concentration of IL-6. Pearson’s Correlation test, *p* = 0.031, R^2^ = 0.5694. (**C**) Serum concentration of IL-6IF according to colon cancer stage. Comparison of stages for probe IL-6IF, *t*-test: Stages I vs. IV (*p* < 0.0001), I vs. III (*p* = 0.0021), I vs. II (NS), II vs. IV (*p* = 0.0027), and II vs. III (*p* = 0.0276). Base signal was compared with all stages with one-way ANOVA + Dunnett’s multiple comparisons test, **** *p* < 0.0001. (**D**) Over-all survival, Kaplan–Meier analysis of colon cancer patients, stage III, showing a significantly reduced survival of patients with a high production of the IL-6IF structure. The discriminatory level was set to 0.7 Logrank test (*p* = 0.0080).

The production of IL-6 by PBMCs in autologous cultures depends on the tumor burden. In preoperative patients, IL-6 production increases from T_2_ to T_3–4_ tumors and further in cultures from patients with lymph node metastases (Figure 4A).

### 3.11. Correlation Between Autoantibody Titer and Serum Concentration IL-6

A possible correlation between the serum concentration of IL-6 and the occurrence of autoantibodies against the IL-6IF structure was investigated. The serum concentrations of IL-6 and autoantibodies are presented in Table S2. A low concentration of these auto-antibodies is considered to be due to a high in vivo production of the IL-6IF struture binding the antibodies in immune complexes which are eliminated. A low titer of autoantibodies thus indicates a high in vivo exposure to the IL-6IF structure.

As shown in Figure 4B, a significant correlation was found between the titer of the autoantibodies and the IL-6 serum concentration in these colon cancer patients (*p* = 0.031). Thus, the serum titer of autoantibodies was inversely correlated with the serum concentration of IL-6 in colon cancer, indicating that a high titer of autoantibodies blocks the activity of IL-6IF.

### 3.12. Determination of the IL-6IF Structure in Sera, Using Biotinylated IL-6IF Peptide as a Probe (Probe IL-6IF)

Next, an inhibition ELISA for determination of the IL-6IF structure in sera from healthy controls and cancer patients was set up. Antibodies directed against the IL-6IF structure released from immune complexes by heat inactivation of control sera as described above were bound to protein G-coated ELISA plates. The biotinylated IL-6IF peptide was used as a probe binding to these ELISA plates. Simultaneous incubation with sera containing the IL-6-inducing structure competed with the binding of the probes and reduced their binding, thereby giving a measure of the amount of the IL-6IF structure in serum.

The concentration of the IL-6IF structure competing with the IL-6IF probe is significantly higher in patients with more advanced cancer. This is in good agreement with the higher production of IL-6 in autologous PBMC cultures from patients with more advanced cancer stages (see Figure 4A). The difference in concentration of the IL-6IF structure in colon cancer patients, stadiums I–IV, is shown in Figure 4C. A comparison of stages for the inhibition of probe IL-6IF, *t*-test: stages I versus IV (*p* < 0.0001), I versus III (*p* = 0.0021), I versus II (NS), II versus IV (*p* = 0.0027), and II versus III (*p* = 0.0276).

### 3.13. Prognostic Significance of the IL-6IF Structure in Serum from Colon Cancer Patients—Survival Analysis

Protein G plates were prepared as described above, incubated with the serum samples to be analyzed, and then incubated with the probe. IL-6IF structures present in sera compete with the probe and inhibit its binding to the autoantibodies bound to the protein G plate.

The serum concentration (Table S3) of these neo-structures was significantly enhance, inhibiting the binding of the probe, resulting in low values in sera from colon cancer patients with poor cancer-specific survival. The discriminatory level was set to be 0.7. Eight stage III patients in the high IL-6IF group (low probe-binding group) died from their cancer, and four were still alive after five years. In the group with low IL-6IF, seven stage III patients were still alive after five years. The Kaplan–Meier analysis is shown in Figure 4D. A high serum concentration of IL-6IF was significantly associated with poor survival (*p* < 0.0080, Logrank test).

## 4. Discussion

IL-6 is of major importance in the regulation of the normal immune system and during the pathological function of the immune system, and it is a key player in both hyperstimulation and suppression. In addition to IL-6-related immunosuppression and poor prognosis in most types of cancers, this cytokine has often been found to be involved in resistance to treatment including all non-surgical modalities: chemo-, radio-, and immunotherapy [11]. Currently, there is a great interest in combining check-point inhibitor therapies with various types of IL-6 inhibitors.

The IL-6 serum concentration is found to be associated with poor prognosis of many types of cancer, particularly in later stages. This, however, does not seem to be the case in colorectal cancer [14,15]. The serum concentration of cytokines such as IL-6 depends on the production rate and its distribution, binding to cells, soluble receptors, therapeutic antibodies, and elimination. Consequently, the appearance of cytokines in sera from pathological conditions can be expected only at a relatively high production rate. IL-6 can seriously impact and dysregulate the function of the immune system despite a low serum concentration. There are several reports on the discrepancy between the presence of IL-6 in tumor tissue and serum. A study on Hodgkin’s disease found no correlation between the IL-6 expression in Reed Sternberg cells or leukocytes and the serum concentration [42]. Similarly, the study by Piancatelli et al. did not find any correlation between IL-6 serum concentration and the expression of IL-6 mRNA in colorectal cancer tissue [43].

In this paper, a large amount of IL-6 was found to be produced by PBMCs in cultures from cancer patients. Untreated melanoma and renal cell carcinoma patients with newly detected metastases had, in general, a serum IL-6 concentration within the normal range, but PBMCs from these patients produced large amounts of IL-6. PBMC cultures are a sensitive method to detect an enhanced production of IL-6, also in patients with localized disease, such as preoperative colorectal cancer or radically resected stage III melanoma. In a previous paper, this type of IL-6 production by PBMCs from preoperative colorectal cancer patients was found to be associated with poor over-all survival [26]. A high production of IL-6 by PBMCs from cancer patients was reported previously [24,25]. Similarly, dysregulation of the immune system has been reported for localized breast cancer [29]. Disease-related IL-6 production by PBMCs can be a useful diagnostic method to detect subclinical disease after radical surgery. The identification of such patients for adjuvant treatment would most likely enhance the therapeutic efficacy. Obviously, the determination of IL-6 production by PBMCs in autologous cultures is a more sensitive method to investigate IL-6-related dysregulation of the immune system than just analyzing the serum concentration.

In these in vitro cultures, PBMCs were either programmed in vivo to produce IL-6 or, possibly, stimulated by serum factors in vitro. Interestingly, as demonstrated in this study, sera from cancer patients were found to stimulate IL-6 production by PBMCs from healthy donors, creating a basis for further analyses of such serum factors. IL-6-inducing activity, which was also found in urine from cancer patients, could be adsorbed by PBMCs. Two-dimensional gel electrophoresis of samples before and after this procedure demonstrated binding of albumin, IgG, and β2-microglobulin fragments to the cells, indicating that albumin fragments could have the capacity to induce IL-6 production. In this context, the generation of albumin fragments by proteolytic degradation of albumin in urine has to be considered [44]. However, as demonstrated here, the IL-6-inducing fragments are also present in sera and are thus not the result of urine proteolytic activity.

The hypothesis that albumin neo-structures generated in cancer might have an IL-6-inducing activity was analyzed by incubating tumor homogenates with albumin. This resulted in fragments, which when tested on normal PBMC, were found to induce the production of IL-6. The impact of proteolytic degradation was then confirmed by the incubation of albumin or IgG with recombinant matrix metalloproteinases followed by an analysis of the IL-6-stimulating activity of fragmented albumin on normal PBMCs. These structures were then identified using artificial cell surface chromatography and synthesized.

Interestingly, full-sized normal albumin does not have any known immunomodulatory activity. Thus, it was postulated that fragmentation generated conformational changes resulting in neo-structures with immune cell-binding properties and immunoregulatory capacity.

These results are compatible with the observation that conformational changes in denatured normal proteins such as human serum albumin, ovalbumin, transferrin, and fibronectin expose structures binding to receptors on a monocyte cell line [45,46]. It has furthermore been demonstrated that the binding of the denatured proteins could be efficiently inhibited by several monoclonal antibodies directed to β2-integrins [45].

The IL-6-inducing capacity of an albumin neo-structure constitutes a completely new mechanism for initiating IL-6 synthesis. A new IL-6-inducing factor, IL-6IF, was identified. Its generation in pathological conditions is characterized by enhanced proteolytic activity such as malignant tumors and chronic inflammation and is in good agreement with the high IL-6 production in these conditions. It also fits very well in “The local initiation model” described by Wilkin [47] and Hirano [3] where a tissue injury, including enhanced proteolytic activity, initiates an inflammatory process.

Many albumin sequences with various biological activities were recently summarized [48]. Conformational changes in albumin resulting in neo-structures with the capacity to bind to immune cells and induce production of IL-6 has been described. Shacter et al. [49] reported that polymers of albumin stimulated peritoneal macrophages to secret interleukin-6 and prostaglandin E2 [49]. Several papers describe that albumin modified by glycation has the capacity to induce IL-6 synthesis [50].

IL-6IF was found to be immunogenic resulting in autoantibody production. Consequently, determination of the IL-6IF in sera from healthy controls, cancer patients, and patients with inflammatory diseases has to take into account that this structure to a variable degree is bound in immune complexes. In addition to IL-6IF, antibodies to this structure and immune complexes containing these constituents are involved in the regulation of IL-6 synthesis. These results highlight an additional level of immunoregulation; not only are there regulatory albumin neo-structures, but their activity is further modulated by the presence of autoantibodies. In cancer, the serum titer of such autoantibodies is significantly lower in advanced disease compared to healthy controls presumably due to a high production of the antigens, the albumin neo-structures, including IL-6IF. These antigens bind autoantibodies in immune complexes which are then eliminated. Consequently, a high systemic exposure to IL-6IF correlates with a low concentration of specific autoantibodies.

The autoantibodies can be released from immune complexes by “standard” heat inactivation of serum (56 °C for 30 min). The antigens, the conformationally changed albumin neo-structures, are presumably aggregated by this procedure, which results in a blockade and a reduced number of antibody binding sites and thereby the release of free antibodies. This procedure shows that immune complexes in sera from healthy controls contain significantly more antibodies than sera from advanced cancer patients. In sera from healthy controls with a high titer of free autoantibodies, the immune complexes exist in antibody excess, whereas in advanced cancer with a low titer of free antibodies, a situation with antigen excess prevails. This means that the immunoregulatory function of free albumin neo-structures is exerted when the autoantibody titer is low. Interestingly, “standard” heat inactivation, a procedure frequently used in in vitro immunological experiments, significantly alters the immune status of sera by releasing autoantibodies directed to such immunoregulatory structures.

The impact of the systemic exposure to IL-6IF was further demonstrated by analyzing the correlation between autoantibodies to this structure and the serum concentration of IL-6 in colorectal cancer patients. Interestingly, a low concentration of specific autoantibodies is significantly correlated with a high serum concentration of IL-6. This demonstrates the importance of IL-6IF for the pathological production of IL-6 in vivo. These results are further supported by the observation that the serum concentration of IL-6IF, determined in an inhibition ELISA, gradually increased with the stage of colon cancer and was significantly higher in sera from advanced stages III and IV compared to stages I and II. It was also found that a high serum concentration of IL-6IF significantly correlated with a poor over-all survival of the patients.

As mentioned above, IL-6 is of major importance in the pathogenesis of a large number of severe diseases such as cancer and chronic inflammation. Therefore, multiple attempts have been made to inhibit its pathological activity by antibodies directed to this cytokine or its receptor or by inhibiting the signal transduction pathways by, for example, JAK/STAT (Janus kinase/signal transducer and activator of transcription) inhibitors. While IL-6-mediated trans-signaling can be selectively inhibited by sgp130fc (Olamkicept), effective treatment for the typically uncontrolled pathological overproduction of IL-6 in numerous conditions is still not available.

IL-6 production by PBMCs from healthy donors incubated with the IL-6IF structure can be inhibited by specific autoantibodies directed against this structure. Thus, specific autoantibodies which selectively inhibit the pathological IL-6 production stimulated by a protease-generated albumin neo-structure were identified. Thereby, the specific epitope of a therapeutic, human monoclonal antibody with the capacity to inhibit pathological production of IL-6 was identified.

IL-6 activity in rheumatoid arthritis and Castleman´s disease can be efficiently inhibited by antibodies in contrast to the situation in cancer. Treating hyperstimulation of the immune system, e.g., cytokine storm elicited by immunotherapy using CAR T cells with antibodies directed against IL-6 or its receptor, has been successful. In this situation, IL-6 most likely does not contribute to the control of the disease. In cancer and infectious diseases, however, the situation is more complicated as IL-6 might still be of importance for the immune-mediated disease control. Treatment with antibodies directed against IL-6 or its receptor unselectively block the activity of this cytokine. In contrast, antibodies directed against IL-6IF described here selectively block pathological IL-6 synthesis related to enhanced proteolytic activity in diseases such as cancer. Furthermore, these antibodies are developed based on the specificity of autoantibodies normally present in healthy persons who thereby are protected from a potential pathological effect of IL-6IF. Therapeutic administration of such antibodies should therefore not cause any adverse events. Consequently, a substantial therapeutic advantage arises from the selective blockade of pathological IL-6 production, allowing the crucial functions of IL-6 in regulating the immune system’s normal response to remain intact, thereby contributing significantly to disease control.

## 5. Conclusions

Pathological IL-6 production in chronic inflammation and thereby in cancer is of major importance in poor cancer control.

More sensitive diagnostic methods of pathological IL-6 synthesis in early stages of cancer would enable early therapeutic intervention in order to ward off the detrimental effects of Il-6.

In this paper, IL-6 synthesis by PBMCs in cultures with autologous serum was found to be a more sensitive method to demonstrate pathological IL-6 production than an analysis of the IL-6 serum concentration. IL-6 production by PBMCs could be detected even in early stages and radically resected local disease.

However, in order to take advantage of this improved diagnostics, therapeutic strategies to inhibit IL-6 are needed. Unfortunately, currently available strategies, using antibodies directed to the cytokine, its receptor, or signal transduction pathways have, so far, had limited success.

Further analyses of IL-6-inducing serum factors identified a quite new IL-6-inducing factor, IL-6IF, generated by an enhanced proteolytic degradation of normal serum albumin. This factor is significantly increased in advanced cancer stages and is significantly correlated with cancer-specific survival.

Autoantibodies to IL-6IF occur in a higher concentration in healthy individuals than in advanced cancer patients, who thereby have less efficient protection against pathological IL-6 production. The identification of these antibodies and the corresponding B-cells enables the production of recombinant monoclonal antibodies selectively inhibiting pathological IL-6 production, leaving normal IL-6 regulation and normal function of the immune system intact.

## Figures and Tables

**Table 1:** IL-6 production by PBMCs from patients with radically resected stage III melanoma (MM 0), previously untreated metastatic melanoma (MM1), previously untreated metastatic renal cell carcinoma (RCC1), and primary colorectal cancer (CRC). IL-6 production is compared to that of healthy controls (K). The table display the mean values ± SEM, and *p* values are calculated using *t*-test.

Group	Patients	IL-6 pg/mL	SEM	*t*-Test
K	49	214	85	-
MM 0	30	2444	978	*p* < 0.0001
MM 1	43	3838	1279	*p* < 0.0001
RCC 1	36	4003	935	*p* < 0.0001
K	12	379	133	-
CRC	46	5973	1362	*p* = 0.042

## Data Availability

The original contributions presented in the study are included in the study/Supplementary Materials, and further inquiries can be directed to the corresponding author.

## Supplementary Materials

The following supporting information can be downloaded at: https://www.mdpi.com/article/10.3390/cancers16213588/s1, Figure S1: Generation of the IL-6IF-structure by PBMC in cultures from healthy controls (grey bars) and advanced cancer patients (black bars) supplemented with albumin; Table S1: IL-6 inducing activity in sera (negative for IL-6) from cancer patients, cultured with PBMCs from healthy individuals. None of the control PBMC made detectable amounts of IL-6 when cultured with autologous, normal sera; Table S2: Serum concentration of auto-antibodies directed against the IL-6IF structure and the serum concentration of IL-6; Table S3: Results from inhibition ELISA, using probe IL-6IF, stage II and III colon cancer.
