# Supplementary material for: A New IL-6-Inducing Mechanism in Cancer with New Therapeutic Possibilities
Source: Cancers (Basel). 2024 Oct 24;16(21):3588. doi: 10.3390/cancers16213588 (PMC11545478; doi:10.3390/cancers16213588)
Supplement: Supplementary file 1 [file cancers-16-03588-s001.zip › cancers-3227073-supplementary.pdf]

Supplemental information

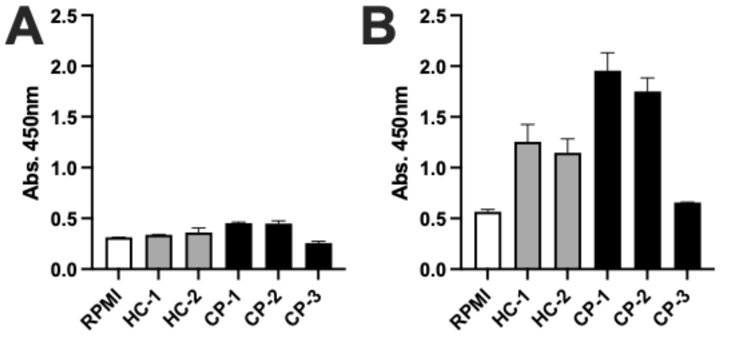

Figure S1. Generation of the IL-6IF-structure by PBMC in cultures from healthy controls (grey bars) and advanced cancer patients (black bars) supplemented with albumin. Enhanced amount of this neo-structure was generated in cultures from cancer patients compared to healthy controls. Increasing amounts of albumin (HSA) was added to the cultures, 0.5 μg/ml (A) and 5μg/ml (B).

Table S1. IL-6 inducing activity in sera (negative for IL-6) from cancer patients, cultured with PBMCs from healthy individuals. None of the control PBMC made detectable amounts of IL-6 when cultured with autologous, normal sera.

Sera from	IL-6, pg/ml	Sera from	IL-6, pg/ml
Patient 1	13129	Patient 6	115
Patient 2	2440	Patient 7	31.2
Patient 3	5313	Control 1	31.2
Patient 4	4951	Control 2	31.2
Patient 5	25276	Control 3	31.2

Table S2. Serum concentration of auto-antibodies directed against the IL-6IF structure and the serum concentration of IL-6.

Sera No.	IL-6 (pg/mL)	IL-6IF AutoAb
G01	14.6	1.670
G02	3.1	2.732
G03	6.5	2.324
G04	12.7	2.530
G05	4.8	2.613
G06	5.6	3.273
G07	22.0	2.138
G08	5.4	3.175
G09	41.3	1.887
G10	3.7	2.911

Table S3. Results from inhibition ELISA, using probe IL-6IF, stage II and III colon cancer.

Patient No	Tumour stage	Inhibitory units	Patient No	Tumour stage	Inhibitory units
C21	2	1,32	C41	3	0,973
C22	2	0,975	C42	3	0,768
C23	2	1,671	C43	3	0,695
C24	2	0,909	C44	3	0,737
C25	2	1,015	C45	3	0,601
C26	2	1,019	C46	3	0,628
C27	2	0,933	C47	3	0,778
C28	2	0,949	C48	3	0,705
C29	2	1,187	C49	3	0,616
C30	2	1,017	C50	3	0,695
C31	2	0,803	C51	3	0,933
C32	2	0,779	C52	3	0,712
C33	2	0,778	C53	3	0,625
C34	2	0,652	C54	3	0,607
C35	2	0,944	C55	3	0,568
C36	2	0,663	C56	3	0,617
C37	2	0,873	C57	3	0,662
C38	2	0,616	C58	3	0,67
C39	2	0,868	C59	3	0,643
C40	2	0,75	C60	3	0,796

Data availability statement

The original contribution presented in the study are included in the article / supplementary material, further inquiries can be directed to the corresponding author.
